# Sex-Specific Interrelationship Between Sleep Quality and Daytime Sleepiness in Predicting Injury Occurrence in Physically Active University Students

**DOI:** 10.3390/jcm15010111

**Published:** 2025-12-23

**Authors:** Jarosław Domaradzki

**Affiliations:** Department of Biological Principles of Physical Activity, Wroclaw University of Health and Sport Sciences, 51-612 Wroclaw, Poland; jaroslaw.domaradzki@awf.wroc.pl

**Keywords:** sleep quality, daytime sleepiness, injury risk, sex differences, fatigue-related impairment, interaction modeling, musculoskeletal injuries, physically active young adults

## Abstract

**Background/Objectives**: Sleep quality and daytime sleepiness influence vigilance and motor control, but their joint contribution to injury risk in physically active young adults remains unclear. This study examined sex-specific associations between sleep quality, daytime sleepiness, and injury occurrence in university students. **Methods**: A cross-sectional sample of 418 students (199 males, 219 females) was analyzed. Sleep quality (PSQI), daytime sleepiness (ESS), and 12-month injury occurrence were assessed with validated questionnaires. Bivariate χ^2^ tests examined individual associations. Sex-stratified log-linear models evaluated classical (multiplicative) interactions between sleep quality (SQ), excessive daytime sleepiness (EDS), and injury (INJ). Additive interaction was assessed using Poisson-derived risk ratios (RR_10_, RR_01_, RR_11_) and synergy indices (RERI, AP, S). **Results**: Poor sleep quality was significantly associated with injury occurrence (χ^2^ = 4.76, *p* = 0.029; OR = 1.60, 95% CI: 1.05–2.45), driven primarily by females (χ^2^ = 5.39, *p* = 0.020; OR = 1.98). In males, interaction plots showed non-parallel slopes and log-linear modeling supported significant two-way dependencies (ΔG^2^ = 18.37, *p* < 0.001), but the three-way interaction was not significant (*p* = 0.119). In females, relationships were fully additive (ΔG^2^ = 0.011, *p* = 0.917). Additive interaction metrics indicated no synergy in males, whereas females showed a mild supra-additive pattern (RR_11_ = 1.61). Importantly, logistic regression models showed that sleep factors explained only 0.6–1.2% of variance in males and up to 4.3% in females, indicating limited overall predictive value. Poor sleep quality contributed modestly to injury occurrence, while daytime sleepiness added minimal explanatory improvement. **Conclusions**: Sleep–injury relationships were sex-specific. Poor sleep quality was the most consistent predictor of injury—especially among females—while interaction patterns differed between sexes. Sleep factors contributed modestly to injury risk and should be interpreted within a broader framework of intrinsic determinants in physically active young adults.

## 1. Introduction

Excessive daytime sleepiness (EDS) is a common yet often overlooked problem among university students [1,2]. Academic stress, irregular schedules, and poor sleep hygiene contribute to disrupted sleep–wake patterns and impaired alertness during the day [3]. Studies have shown that between 20% and 40% of students report symptoms of EDS, which are associated with reduced academic performance, decreased attention, and a higher likelihood of risky behaviors [4]. Persistent daytime sleepiness also reflects accumulated sleep debt and physiological fatigue, which may compromise psychomotor performance and decision-making in everyday activities.

Sleep quality is a multidimensional construct encompassing sleep duration, latency, efficiency, and perceived restfulness [5]. The Pittsburgh Sleep Quality Index (PSQI) is a widely used instrument for assessing global sleep quality, with scores above 5 typically indicating poor sleep quality and clinically relevant disturbances [6]. Among university students, the prevalence of poor sleep quality often exceeds 50%, reflecting the combined influence of academic workload, stress, and social habits [7,8] and sleep quality in young adults has been linked to emotional dysregulation, impaired cognitive performance, and decreased academic achievement [9,10]. Environmental and behavioral factors, such as late-night electronic media use and irregular sleep schedules, further exacerbate sleep fragmentation and subjective fatigue [11].

Daytime sleepiness reflects a decreased ability to maintain wakefulness and alertness during typical waking hours [12]. It arises from an imbalance between homeostatic sleep pressure and circadian regulation, often resulting from insufficient or poor-quality nocturnal sleep [13]. Physiologically, excessive daytime sleepiness (EDS) is characterized by reduced cortical arousal and slower neuronal responsiveness, leading to diminished attention, delayed reaction time, and lapses in cognitive performance [14,15]. Psychologically, EDS manifests as difficulty concentrating, irritability, and transient “microsleeps” that interfere with learning and task execution [16]. In young adults, common behavioral contributors include irregular sleep–wake schedules, late-night screen exposure, caffeine misuse, and social jet lag [17,18]. The Epworth Sleepiness Scale (ESS) is widely used to quantify subjective sleepiness, with scores ≥ 11 typically indicating clinically relevant EDS. Persistent daytime sleepiness in students, therefore, represents both a symptom of underlying sleep dysregulation and a potential precursor to performance errors and accidental injuries [19].

Young adults, especially those engaged in regular physical activity, represent a group with relatively high exposure to unintentional injuries [20]. Although these injuries are usually minor, they may have significant consequences for well-being and academic participation [21]. Impaired vigilance, slowed reaction times, and fatigue-related attentional lapses are well-documented risk factors for accidental injuries, both in occupational and athletic contexts [22,23]. Epidemiological studies have also reported that insufficient sleep duration and excessive daytime sleepiness are associated with an increased likelihood of traffic, work, and daily-life accidents in young adults [24,25]. However, few studies have specifically examined how sleep-related factors contribute to injury risk in non-athletic but physically active student populations, which is a context that bridges everyday activity and recreational exercise [8].

Although poor sleep quality and daytime sleepiness are correlated, they represent distinct aspects of sleep–wake functioning [12]. Poor sleep quality primarily reflects disturbances in nocturnal rest, such as prolonged latency, frequent awakenings, and nonrestorative sleep, whereas daytime sleepiness indicates the residual consequences of insufficient or fragmented sleep on alertness and wake functioning [15]. Both factors may independently increase the risk of injuries through impaired vigilance, slowed reaction times, and reduced motor coordination [19]. Experimental and observational studies have shown that concurrent poor sleep and elevated sleepiness lead to additive or even synergistic effects on accident risk, particularly in transportation and occupational settings [22,26]. However, few studies have explicitly examined this combined relationship in young, physically active populations such as university students, where both factors are prevalent yet rarely studied together [7,17]. Investigating the interactive effect of poor sleep quality and excessive daytime sleepiness could, therefore, clarify whether their coexistence magnifies the risk of unintentional injury beyond their individual contributions.

Most previous research on sleep-related injury risk has focused either on sleep quality or daytime sleepiness as isolated determinants of health and performance outcomes, rather than examining their combined effects [25,26]. Studies among students and young adults have linked poor sleep quality to academic difficulties, stress, and decreased attention, while excessive daytime sleepiness has been associated with fatigue and impaired reaction times [4,10]. However, research simultaneously considering both dimensions—and especially testing their interaction in relation to unintentional injury risk—remains scarce [17]. To date, the majority of interaction studies have been conducted in professional or clinical populations (e.g., shift workers, drivers, or patients with sleep disorders), not in university students, who frequently experience chronic sleep restriction and irregular circadian rhythms [23,24].

This study, therefore, addresses a clear research gap by jointly examining sleep quality (PSQI) and daytime sleepiness (ESS) as co-occurring risk factors for injury in a non-clinical, physically active university population, and by statistically testing whether their combined or interactive effect amplifies injury risk beyond individual contributions.

The novelty of this approach lies in its integrative modeling of two complementary sleep dimensions—nighttime rest and daytime alertness—to identify high-risk sleep phenotypes within a young, health-oriented cohort. This study extends existing evidence by focusing on university students—physically active young adults outside elite sport and clinical contexts—where research combining sleep quality (PSQI) and daytime sleepiness (ESS) in relation to injury risk remains scarce. By formally testing their interaction and additive synergy (RERI, AP, S), the study advances understanding beyond single-factor analyses and highlights practical implications for sleep hygiene education, early screening of excessive daytime sleepiness, and injury prevention in academic settings.

The main aim of this study is to investigate the independent and interactive effects of sleep quality and daytime sleepiness on the risk of unintentional injury among university students. Both sleep quality and sleepiness are known to impair vigilance, psychomotor coordination, and executive functioning [3,19], but their combined contribution to injury risk in young adults has not been systematically explored [26]. Specifically, this work aims to (1) estimate the individual associations of poor sleep quality (PSQI) and excessive daytime sleepiness (ESS) with injury occurrence; (2) study the potential interaction between sleep quality and daytime sleepiness in predicting injury risk, assessing the nature of their interrelationship using both multiplicative and additive interaction models; (3) examine sex-specific differences in the dependence structure linking sleep quality, daytime sleepiness, and injury occurrence; and (4) evaluate the relative contribution of sleep quality and daytime sleepiness to injury occurrence, and to contextualize their effects within the broader spectrum of intrinsic risk factors in physically active young adults.

Accordingly, the following hypotheses were formulated: H1. Poor sleep quality and excessive daytime sleepiness are each independently associated with a higher risk of injury in physically active university students. H2. Sleep quality and daytime sleepiness interact in predicting injury risk, such that their combined effect deviates from what would be expected based on their individual contributions (multiplicative or additive interaction). H3. The dependence structure linking sleep quality, daytime sleepiness, and injury occurrence differs between males and females, reflecting sex-specific patterns of interaction. H4. Sleep-related factors (sleep quality and daytime sleepiness) explain only a portion of injury risk, and their contribution is expected to be modest relative to other intrinsic risk factors common in physically active young adults (e.g., movement quality, postural characteristics, and body composition).

## 2. Materials and Methods

For the present study, datasets from two independent cohorts of university students were merged to ensure an adequate sample size for the planned analyses on sleep-related predictors of injury risk. Data collection was carried out between 2022 and 2023 and encompassed anthropometric evaluations, body composition and balance testing, as well as a comprehensive set of self-reported questionnaires addressing injury occurrence, lifestyle behaviors (dietary patterns, physical activity), sleep characteristics, mental health, quality of life, and socio-economic background. The final sample size differs from that described in previous reports, as only participants with complete information on variables relevant to the current analyses were retained, and the two cohorts were initially recruited and assessed separately.

### 2.1. Study Design

The present study employed a cross-sectional design with a convenience sampling approach among university students. Data were collected between 2022 and 2023 at the Wroclaw University of Health and Sport Sciences from students enrolled in physical education, sport, and physiotherapy programs. Participants first completed an online questionnaire including sections on injury occurrence, sleep habits, daytime sleepiness, and general lifestyle characteristics. This was followed by anthropometric and body composition assessments conducted under standardized laboratory conditions.

Data originating from two independent student cohorts were merged to ensure sufficient statistical power for analyses focused on sleep-related factors. The main outcome variable was injury occurrence (INJ), while sleep quality (SQ) and daytime sleepiness (EDS) served as key explanatory variables. Given the observed sex-specific patterns in the preliminary analyses, all subsequent statistical procedures were conducted separately for males and females. Therefore, sex was not included as a covariate. The analytical framework focused on evaluating individual and interactive effects of sleep quality and daytime sleepiness on injury risk within each sex group.

### 2.2. Ethics

The study protocol received ethical approval from the Senate Research Ethics Committee of the Wroclaw University of Health and Sport Sciences (approval no. 13/2022). All procedures were conducted in accordance with the principles of the Declaration of Helsinki. Prior to participation, students were provided with detailed information about the study aims, procedures, and data confidentiality. Informed consent was obtained electronically from each participant before data collection commenced.

### 2.3. Sample Size

The target sample size was determined according to commonly accepted guidelines for multivariable epidemiological analyses. For logistic regression, the conventional rule of thumb of at least ten outcome events per predictor was used as a heuristic benchmark to ensure stable estimation of model parameters and interaction terms [27,28,29]. As exploratory analyses were planned—including stratified models, interaction testing between sleep quality and daytime sleepiness, and additional procedures such as log-linear modeling and synergy assessment (RERI, AP, S)—a relatively large sample was required to maintain stable estimates across subgroups. To complement this heuristic approach, a formal sample size calculation was performed using the standard formula for estimating a population proportion with a specified margin of error (δ) at a 95% confidence level [30,31]:

$$n=(\frac{1.96}{\delta }{)}^{2}\times p\left(1-p\right).$$

Assuming a small margin of error (δ = 0.05) and maximum variance (*p* = 0.5), the required sample size was 385. To account for possible nonresponse and incomplete data, an additional 20% was added, yielding a final target of approximately 460 participants. These conservative assumptions provided sufficient power for subgroup comparisons by sex and for interaction testing between sleep quality and daytime sleepiness. For subgroup analyses that did not meet the “10 cases per predictor” guideline, this rule was treated as a heuristic benchmark. To reduce potential bias in small-sample models, supplementary sensitivity analyses using Firth’s bias-reduced logistic regression were performed [32].

### 2.4. Participants

**Figure 1:**
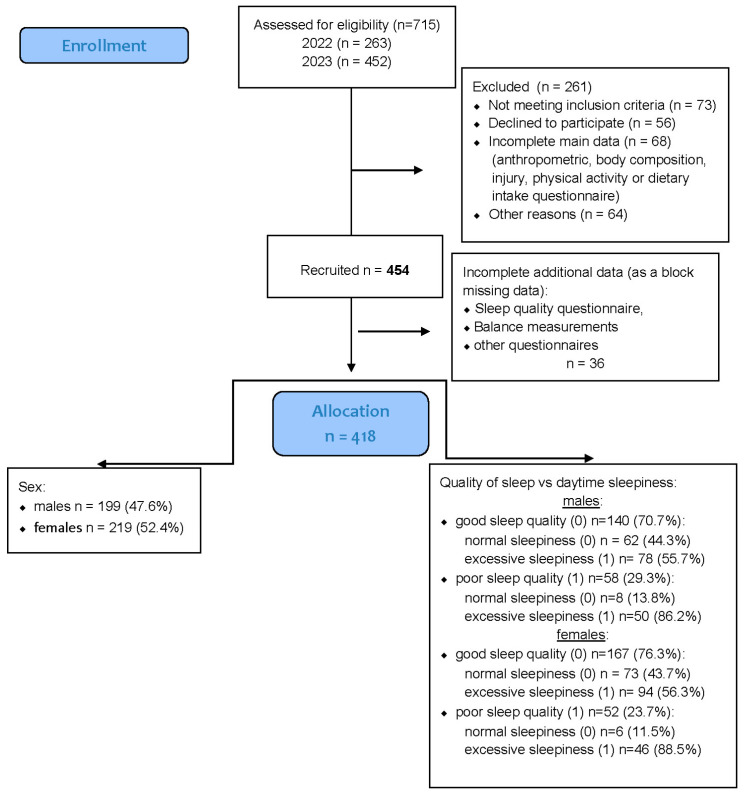
Flow diagram of the progress through all phases of data collection.

A total of 454 healthy university students were recruited for the study, comprising 219 men (48%) and 235 women (52%). All participants were enrolled in the Faculty of Physical Education, Sport, and Physiotherapy at the Wroclaw University of Health and Sport Sciences during the 2022–2023 academic year. Recruitment was conducted among students attending regular in-person classes, and participation was voluntary. The sex distribution within the sample closely reflected that observed in these study programs. A detailed overview of the recruitment and data inclusion process is provided in Figure 1.

Preliminary inclusion criteria required participants to be physically active students who regularly attended in-person university classes. Individuals engaged in university-level competitive sports or enrolled in specialized athletic or elite performance programs were excluded to ensure sample homogeneity with respect to habitual physical activity.

A total of 454 students initially met the inclusion criteria and were considered eligible for participation. All provided complete anthropometric data and valid responses for the sleep quality (PSQI), daytime sleepiness (ESS), and injury history questionnaires.

However, 36 participants were excluded due to missing data in key variables (sleep quality, sleepiness, or injury occurrence), which were treated as block-missing cases.

An additional 13 participants had incomplete responses limited to a single variable (e.g., PSQI or ESS score); these data were handled through multiple imputations, as described in Section 2.7.

After data cleaning and imputation procedures, the final analytical sample consisted of 418 participants. Mean total physical activity levels, expressed in MET·min/week, indicated that both males (3608 ± 1357) and females (3019 ± 1001) exhibited, on average, high levels of physical activity according to the IPAQ classification (≥3000 MET·min/week threshold).

### 2.5. Anthropometric Measurements

Anthropometric assessments were performed in the Biokinetics Research Laboratory, a division of the Central Research Laboratory at the Wroclaw University of Health and Sport Sciences. The facility operates under a certified Quality Management System compliant PN-EN ISO 9001:2015 (Certificate No. PW-15105-22X) [33].

Body height was measured twice to the nearest 0.1 cm using a GPM anthropometer (GPM Instruments GmBH, Susten, Switzerland). Body mass and body fat percentage were assessed using bioelectrical impedance analysis (BIA) with the InBody 230 analyzer (InBody Co. Ltd., Cerritos, CA, USA). Measurements were taken in light sportswear and without shoes, following manufacturer guidelines. Based on these data, the Body Mass Index (BMI) was calculated according to the following formula:

$$BMI=\frac{body\;mass\;[\mathrm{kg}]}{body\;height{\;[m}^{2}]}$$

### 2.6. Questionnaire Measurements

#### 2.6.1. Sleep Quality—Pittsburgh Sleep Quality Index (PSQI)

Sleep quality was assessed using the Pittsburgh Sleep Quality Index (PSQI) [5], a widely validated self-report questionnaire evaluating habitual sleep patterns over the previous month. The instrument consists of 19 items grouped into seven components: subjective sleep quality, sleep latency, sleep duration, habitual sleep efficiency, sleep disturbances, use of sleep medication, and daytime dysfunction.

Each component is scored on a 0–3 scale, yielding a global PSQI score ranging from 0 to 21, where higher values indicate poorer sleep quality. In this study, participants with scores ≤ 5 were classified as having good sleep quality, whereas those with scores ≥ 6 were classified as having poor sleep quality, consistent with standard cut-off criteria used in sleep research.

#### 2.6.2. Daytime Sleepiness—Epworth Sleepiness Scale (ESS)

Daytime sleepiness was measured using the Epworth Sleepiness Scale (ESS), a self-administered instrument assessing the general propensity to fall asleep in eight everyday situations, such as reading, watching television, or sitting in a car. Respondents rate their likelihood of dozing off in each situation on a 4-point Likert scale (0–3), producing a total score between 0 and 24. Higher scores denote greater sleepiness. A value of ≥11 points was used to define excessive daytime sleepiness (EDS), indicating increased sleep propensity and potential impairment of daytime functioning. The ESS is a validated and reliable screening tool commonly employed in both clinical and non-clinical sleep studies.

#### 2.6.3. Injury Occurrence—Injury History Questionnaire (IHQ)

Injury occurrence during the previous 12 months was assessed using a standardized Injury History Questionnaire (IHQ) developed for epidemiological research on physically active populations [34]. Participants reported the number and type of injuries sustained during sports, recreational, or daily activities, along with the body region affected and time loss (if applicable). For analytical purposes, injury data were dichotomized as injured (1) or non-injured (0), representing the primary outcome variable. The IHQ has demonstrated satisfactory test–retest reliability in studies involving young adults and student populations.

#### 2.6.4. Physical Activity—International Physical Activity Questionnaire (IPAQ)

Physical activity was assessed using the Polish adaptation of the International Physical Activity Questionnaire—Long Form (IPAQ-LF) [35], administered electronically via Google Forms.

### 2.7. Handling and Imputation of Missing Data

Missing data were identified for sleep quality (PSQI) in six cases, daytime sleepiness (ESS) in four cases, and injury history (IHQ) in three cases. Because multivariable analyses required complete datasets, missing values were imputed prior to statistical modeling. The missing data mechanism was examined using Little’s MCAR test, which indicated that the pattern of missingness could be classified as Missing Completely at Random (MCAR) [36,37], suggesting no systematic bias related to participant characteristics or study variables. Imputation was conducted in R (RStudio 2025.09.1+401) using the mice package (v3.14.0; accessed 15 October 2024).

### 2.8. Statistics

All statistical analyses were performed using Statistica 14.0 (TIBCO Software Inc., Palo Alto, CA, USA) and RStudio v. 2025.09.1+401 (Posit team (2025). RStudio: Integrated Development Environment for R. Posit Software, PBC, Boston, MA. USA) with the mice (version 3.19.0), epiR (version 2.0.89), and interaction R (version 0.1.7) packages. Prior to analysis, all continuous variables were checked for normality using the Shapiro–Wilk test and for homogeneity of variances with Levene’s test.

#### Design of Analysis (DoA)

Baseline descriptive statistics were summarized as means ± standard deviations or frequencies (%), depending on the measurement scale. Sex differences in baseline values of continuous variables (age, anthropometric measures, and sleep scores) were assessed using independent-samples *t*-tests.

Associations between sleep factors and injury occurrence—χ^2^ independence test. Associations between categorical baseline variables were examined using the Chi-square (χ^2^) test of independence. This included cross-tabulations of sex × sleep quality (good vs. poor) and sex × daytime sleepiness (normal vs. excessive) to verify whether the distribution of male and female participants differed across sleep-quality and sleepiness categories. For each contingency table, we reported the χ^2^ statistic, degrees of freedom, *p*-value, and phi (φ) or Cramer’s V coefficient as a measure of association strength. Expected frequencies were inspected to confirm the validity of χ^2^ assumptions. When expected cell counts were below 5, Fisher’s exact test was applied instead.

Multiplicative interaction—log-linear modeling. To examine dependence structures and potential interactions among the categorical variables, log-linear models were fitted to the three-way contingency table defined by INJ (0/1) × SQ (0/1) × EDS (0/1). A hierarchical modeling strategy was applied, beginning with models including all main effects, then sequentially adding two-way interactions, and finally testing the three-way interaction term. Model comparisons were performed using likelihood-ratio tests (LRT). The SQ × EDS interaction was the pre-specified effect of primary interest. Model fit was evaluated using residual deviance and information criteria (AIC/BIC). Where appropriate, fitted cell counts and interaction estimates were translated into interpretable measures of association within relevant strata.

Additive (synergistic) interaction—RERI/AP/S analysis. To quantify additive interaction between sleep quality and daytime sleepiness within each sex, we estimated the Relative Excess Risk due to Interaction (RERI), the Attributable Proportion due to interaction (AP), and the Synergy Index (S), each with corresponding 95% confidence intervals (delta method or bootstrapping). These indices were derived from risk ratios (RR) estimated using a Poisson regression model with robust standard errors [38], fitted separately for males and females:

INJ ~ SQ + EDS + SQ×EDS; link = log

This modeling approach was chosen because Poisson regression with robust standard errors provides a stable and widely recommended method for deriving risk ratios needed for RERI, AP, and S in cross-sectional datasets with non-rare binary outcomes. GEE models were not applied because the data lacked clustered or repeated structures, and GEE-derived coefficients do not directly yield risk ratios suitable for computing additive interaction metrics. Full bootstrapping was also avoided, as the moderate overall sample size—and especially the small sex-specific subgroups—would likely produce unstable interaction estimates. Therefore, Poisson regression with robust SE was the most appropriate and parsimonious option for the present analytical framework, although the resulting interaction estimates should be interpreted as exploratory due to sample-size limitations.

Positive values (RERI > 0, AP > 0, S > 1) indicate positive additive interaction (synergy).

Statistical assessment of the contribution of sleep-related predictors. To quantify the explanatory power of sleep-related predictors, sex-stratified logistic regression models were fitted, comparing incremental model fit across nested models (SQ only; EDS only; SQ + EDS; SQ × EDS). Model performance was evaluated using likelihood-ratio χ^2^ tests against the null model, Nagelkerke R^2^ as an index of variance explained, and information criteria (AIC, BIC). This allowed estimation of the relative contribution of sleep quality and daytime sleepiness to overall injury risk in males and females.

All analyses were adjusted for sex, age, and BMI. Statistical significance was set at *p* < 0.05. Graphical visualizations of the interaction effects were created using ggplot2 in R.

Generative AI tools were used in accordance with COPE and MDPI transparency principles. Their role was strictly limited to preparatory and editorial support. Chat Academia (v.1.0, 2025) assisted in refining research questions and identifying potential gaps, while Scholarcy (v.4.0, 2025) and NotebookLM (v.1.3, 2025) were used only to generate preliminary summaries and reading notes for selected articles. Elicit (v.2.0, 2025) supported semantic literature search and identification of methodological patterns. For the statistical workflow, ChatGPT (OpenAI, GPT-4.1, 2025) assisted solely in locating documentation for R packages (including CRAN Task Views and Scikit-lmm). No AI tool was used to conduct statistical analyses, generate results, interpret findings, or draw scientific conclusions. Minor R-code errors were corrected using AI-assisted debugging tools within RStudio, followed by manual verification. ChatGPT was also used during manuscript preparation for preliminary drafting and language refinement. All AI-assisted content was reviewed, corrected, and approved by the author, who assumes full responsibility for the final manuscript.

## 3. Results

### 3.1. Participant Characteristics

Baseline characteristics are presented in Table 1. Descriptive data are shown as means ± standard deviations and 95% CI for means. Anthropometric characteristics differed significantly between males and females, with males exhibiting higher values across all somatic measures (all *p* < 0.05). Similarly, males were significantly more physically active than females (*p* < 0.001), although both groups exceeded the 3000 MET·min/week threshold, indicating a generally highly active sample. In contrast, no sex differences were observed in sleep quality (PSQI: *p* = 0.431) or excessive daytime sleepiness (EDS: *p* = 0.437), indicating that the quality of sleep and daytime sleepiness were comparable between male and female participants.

Among the 418 participants, 199 (47.6%) were male, and 219 (52.4%) were female. Injuries were reported by 56.8% of males and 46.6% of females (Table 2). Males more often suffered from at least one injury (56.8%) compared to females (46.6%). Based on sleep quality, 280 participants (67.0%) were classified as good sleepers and 138 (33.0%) as poor sleepers. Among good sleepers, 47.5% reported at least one injury, whereas among poor sleepers, this proportion increased to 59.4%. With respect to daytime sleepiness, 138 participants (33.0%) demonstrated normal levels of daytime alertness, while 280 participants (67.0%) reported excessive daytime sleepiness. The injury rate was higher among those with excessive sleepiness (53.6%) compared with participants with normal daytime sleepiness (47.1%) (Table 2).

### 3.2. Bivariate Associations Between Sleep-Related Factors and Injury Occurrence

Chi-square analyses indicated that injury occurrence did not differ significantly between sexes (χ^2^(1) = 0.57, *p* = 0.453), although males showed a slightly higher injury rate than females (OR = 1.16, 95% CI: 0.79–1.69; φ = 0.04) (Table 3). Sleep quality and daytime sleepiness likewise did not differ by sex (both *p* > 0.55).

Poor sleep quality was significantly associated with higher injury occurrence (χ^2^(1) = 4.76, *p* = 0.029), with poor sleepers showing 1.60 times higher odds of injury compared with good sleepers (95% CI: 1.05–2.45; φ = 0.11). Excessive daytime sleepiness was not significantly related to injury occurrence (χ^2^(1) = 1.24, *p* = 0.265), although the effect size indicated a mild, non-significant elevation in risk among sleepy participants (OR = 1.27, 95% CI: 0.83–1.95; φ = 0.06) (Table 3).

Sex-stratified analyses revealed different patterns across males and females.

Among males, neither sleep quality nor daytime sleepiness predicted injury occurrence (both *p* > 0.40).

Among females, poor sleep quality showed a statistically significant association with injury risk (χ^2^(1) = 5.39, *p* = 0.020), with poor sleepers demonstrating 1.98-fold higher odds of injury (95% CI: 1.11–3.56; φ = 0.16). Daytime sleepiness was not significantly associated with injuries among females (*p* = 0.148), although the effect size suggested a possible trend (OR = 1.47, 95% CI: 0.86–2.51; φ = 0.10) (Table 3).

Overall, poor sleep quality emerged as the sleep-related factor most strongly linked to injury occurrence, particularly among female participants. To evaluate whether sleep quality and daytime sleepiness may interact in shaping injury risk, a log-linear analysis was conducted in the next step.

### 3.3. Log-Linear Analysis of the Multiplicative (Classical) Interaction Effects

To examine whether sleep quality (SQ) and excessive daytime sleepiness (EDS) jointly influence injury occurrence beyond their independent effects, we fitted hierarchical log-linear models to the 2 × 2 × 2 contingency structure defined by SQ × EDS × injury status (INJ). This approach evaluates classical (multiplicative) interaction, meaning that the combined effect of SQ and EDS on injury risk deviates from what would be expected under a strictly multiplicative model of association.

**Figure 2:**
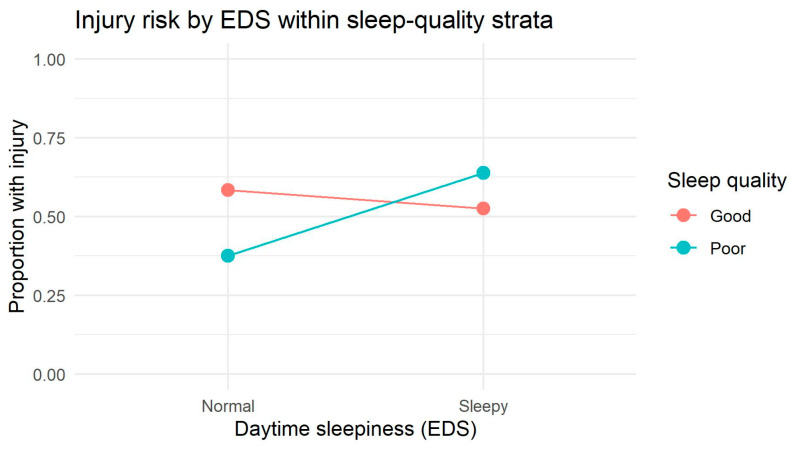
Injury risk as a function of daytime sleepiness stratified by sleep-quality categories in males. The figure presents proportions of participants reporting an injury in groups defined by daytime sleepiness (normal vs. sleepy) within each sleep-quality category (good vs. poor sleep). Lines are not parallel, suggesting a descriptive interaction pattern; however, the corresponding three-way interaction (SQ × EDS × INJ) was not statistically significant in log-linear modeling.

The interaction plot for males showed visually diverging, crossing slopes between sleep-quality strata, indicating a clear qualitative pattern consistent with potential interaction (Figure 2). This graphical pattern aligns with the significant improvement when two-way interactions were included, confirming that sleep quality and daytime sleepiness jointly relate to injury occurrence in males.

For males, the main-effects log-linear model showed a significant lack of fit (G^2^ = 20.79, df = 4, *p* < 0.001), demonstrating that at least some associations among sleep quality, daytime sleepiness, and injury occurrence were present within this subgroup. Introducing all two-way interactions markedly improved model fit (G^2^ = 2.42, df = 1, *p* = 0.119), and the likelihood-ratio comparison against the main-effects model was highly significant (ΔG^2^ = 18.37, Δdf = 3, *p* = 0.00037). This indicates that the dependence structure among sleep quality, daytime sleepiness and injuries in males is fully accounted for by pairwise associations. Adding the three-way interaction term (SQ × EDS × INJ) did not improve the model further (ΔG^2^ = 2.42, Δdf = 1, *p* = 0.119), suggesting no statistically significant multiplicative three-way interaction.

These results indicate that among males, sleep quality and daytime sleepiness relate to injury occurrence primarily through significant two-way dependencies, while showing a visible but statistically non-significant trend toward a higher-order multiplicative interaction. Overall, the pattern is more consistent with a predominantly additive interaction structure, but with a suggestive tendency toward multiplicative effects, matching the non-parallel lines observed in the interaction plot (Table 4).

**Figure 3:**
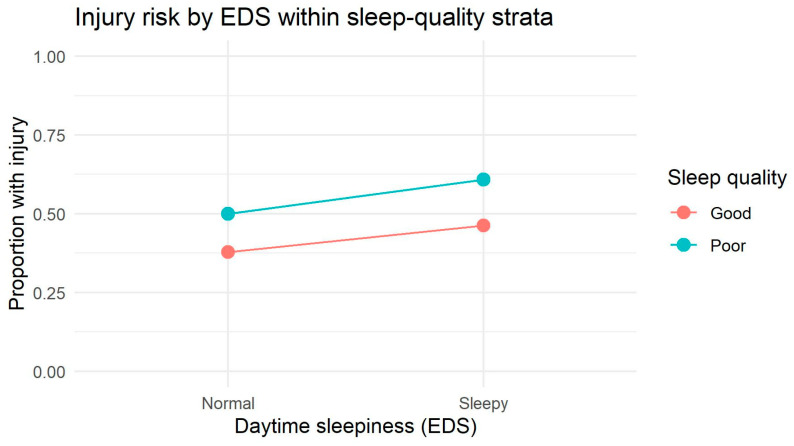
Injury risk as a function of daytime sleepiness stratified by sleep-quality categories in females. The figure presents proportions of participants reporting an injury in groups defined by daytime sleepiness (normal vs. sleepy) within each sleep-quality category (good vs. poor sleep). Lines are not parallel, suggesting a descriptive interaction pattern; however, the corresponding three-way interaction (SQ × EDS × INJ) was not statistically significant in log-linear modeling.

In contrast to the male subgroup, the lines in the interaction plot (Figure 3) remained essentially parallel across sleep-quality strata, indicating that the effect of daytime sleepiness on injury occurrence was additive and did not differ as a function of sleep quality. This visual pattern is fully consistent with the log-linear results, which showed no evidence of a two- or three-way (SQ × EDS × INJ) interaction in this subgroup.

For females, the main-effects model showed a clear lack of fit (G^2^ = 27.08, df = 4, *p* < 0.001), indicating that significant associations existed among sleep quality, daytime sleepiness, and injury occurrence in this subgroup (Table 5). As with males, adding all two-way interactions dramatically improved model fit (G^2^ = 0.011, df = 1, *p* = 0.917), and the likelihood-ratio comparison against the main-effects model was highly significant (ΔG^2^ = 27.07, Δdf = 3, *p* < 0.00001). This demonstrates that pairwise dependencies fully captured the relationships between SQ, EDS, and INJ among females. Introducing the three-way interaction term (SQ × EDS × INJ) did not further improve model fit (ΔG^2^ = 0.011, Δdf = 1, *p* = 0.917), providing no evidence of a multiplicative three-way interaction in this subgroup.

These findings suggest that among females, sleep quality and daytime sleepiness relate to injury occurrence through stable and consistent two-way effects, and there is no indication—either statistical or visual—of higher-order multiplicative interaction. The pattern is therefore one of a pure additive structure, with sleep-related factors influencing injury risk independently rather than synergistically.

### 3.4. Additive Interaction (Synergy) Analysis

To evaluate whether the joint occurrence of poor sleep quality (SQ) and excessive daytime sleepiness (EDS) produced a combined effect on injury risk exceeding the sum of their individual contributions, an additive interaction analysis was performed. Unlike the log-linear approach, which tests multiplicative (classical) interactions, the present analysis focused on risk-based interaction metrics, which provide a more appropriate framework for detecting synergistic effects on an additive scale.

Among males, the Poisson regression model indicated a pattern of multiplicative rather than additive risk. The joint exposure did not exceed the expected additive value (RR_11_ ≈ RR_10_ + RR_01_ − 1), and the indices (RERI = 0.55, AP = 0.50, S < 1) were inconsistent and unstable, reflecting the absence of supra-additive synergy. The combined effect remained essentially additive (Table 6).

In contrast, among females, the joint effect of poor sleep quality and excessive daytime sleepiness showed a pattern consistent with a supra-additive interaction. The combined exposure produced a higher risk (RR_11_ = 1.61) than would be expected from the sum of individual effects (expected additive RR = 1.54). Although RERI (0.07), AP (0.04), and S (1.12) were numerically small and confidence intervals included the null, the relative risk structure clearly followed the qualitative definition of a supra-additive pattern.

### 3.5. Relative Contribution of Sleep Quality and Daytime Sleepiness to Injury Occurrence

Logistic regression models showed that sleep-related factors explained only a small proportion of the variance in injury occurrence in both sexes (Table 7).

Among males, all models showed negligible explanatory value, with Nagelkerke R^2^ ≤ 0.012 and non-significant likelihood ratio tests (all *p* ≥ 0.33). Neither sleep quality nor daytime sleepiness meaningfully improved fit over the null model, and adding the SQ × EDS interaction did not increase predictive performance (AIC 278.40 vs. 276.19 in single-factor models). Among females, sleep quality contributed modestly but significantly to injury occurrence (Nagelkerke R^2^ = 0.037; LR χ^2^ = 6.15, *p* = 0.013). Adding daytime sleepiness slightly increased explanatory value (R^2^ = 0.043; LR χ^2^ = 7.11, *p* = 0.029), although the interaction model did not improve fit further (*p* = 0.066). Despite statistical significance, the overall magnitude of explained variance remained low (R^2^ ≤ 0.043).

Overall, sleep variables accounted for only 0.6–1.2% of variance in males and up to 4.3% in females, indicating that sleep quality and daytime sleepiness play a limited but sex-specific role in explaining injury risk.

## 4. Discussion

This study examined the independent and interactive contributions of sleep quality (SQ) and excessive daytime sleepiness (EDS) to the risk of unintentional injury in a large sample of physically active university students. Several important findings emerged. First, poor sleep quality—but not daytime sleepiness—was significantly associated with higher injury occurrence, with this effect driven primarily by female participants. Second, sex-specific interaction patterns were evident: males displayed a descriptive interaction between SQ and EDS, whereas females showed a strictly additive relationship. Third, additive interaction metrics suggested a supra-additive tendency in females and no such pattern in males, although these estimates were small and imprecise. Together, these results highlight substantive sex differences in how sleep-related factors combine to influence injury risk in young, physically active adults. The relative contribution of sleep-related factors to injury risk was modest in this physically active population. Sleep variables demonstrated limited explanatory power, accounting for approximately 1% of variance in injury risk among males and up to 4% among females. Although sleep quality emerged as a significant predictor in women, its overall effect size was modest, and daytime sleepiness added only minimal additional value. These patterns suggest that sleep-related factors represent just one component in a broader constellation of intrinsic determinants of injury risk. Importantly, because the study is cross-sectional, these associations cannot be interpreted as causal, and temporal ordering between sleep characteristics and injury cannot be determined.

A central finding of this study is that sleep quality, rather than daytime sleepiness, emerged as the strongest sleep-related determinant of injury. This aligns with prior evidence showing that poor sleep quality impairs cognitive control, reaction time, psychomotor vigilance, and emotional regulation—mechanisms directly linked to injury susceptibility [14,39]. Poor sleep quality has been associated with higher odds of injury and severe injury, as determined by logistic regression analysis of 192,480 individuals [40]. The particularly robust association observed among females is consistent with literature showing that women report more sleep complaints and show greater physiological sensitivity to sleep disturbances than men [41,42]. Although daytime sleepiness was not independently associated with injuries, the small effect size observed suggests that habitual sleepiness may still function as a secondary risk factor, especially when combined with poor sleep quality. Several neurophysiological mechanisms may explain why poor sleep quality contributes more strongly to injury risk than daytime sleepiness. Fragmented or inefficient sleep disrupts prefrontal cortical functioning, leading to impaired inhibitory control, reduced risk evaluation, and increased impulsive responding—key pathways linked to accidental injury [43]. Moreover, sleep fragmentation alters sensorimotor integration and proprioceptive accuracy, producing delayed neuromuscular responses and reduced postural stability [44,45], which increases susceptibility to missteps and movement errors during physical activity. In addition, chronic sleep disturbance elevates systemic inflammation and pain sensitivity [46], potentially lowering functional thresholds at which minor biomechanical stressors lead to injury.

The sex-specific interaction patterns represent one of the most novel contributions of this study. Among males, the interaction plot revealed visibly non-parallel, crossing slopes between sleep-quality strata, indicating a descriptive tendency toward interaction. Log-linear modeling supported this observation by showing that two-way dependencies significantly improved model fit, although the three-way multiplicative interaction did not reach statistical significance. This suggests that in males, sleep quality and daytime sleepiness jointly influence injury occurrence, but predominantly through additive or near-multiplicative structures rather than a robust synergistic effect. Such patterns may reflect behavioral and physiological differences—males tend to accumulate greater sleep debt, exhibit more variable sleep timing, and demonstrate greater fluctuations in risk-taking and attentional control, especially under fatigue [47,48].

Among females, by contrast, interaction plots showed parallel lines, and both log-linear and additive models confirmed a stable additive structure without evidence of higher-order interaction. This indicates that SQ and EDS exert their effects on injury risk independently. Such findings are consistent with research showing that females maintain more regular sleep patterns, have greater sleep efficiency, and may rely more heavily on compensatory regulatory mechanisms that dampen the combined impact of multiple sleep disruptions [49,50]. Nonetheless, the additive interaction analysis revealed a mild supra-additive tendency in females (RR_11_ > RR_10_ + RR_01_ − 1), suggesting that qualitative interaction may occur even in the absence of significant statistical interaction. This underscores the importance of complementing multiplicative tests (log-linear models) with risk-difference-based metrics [38].

Several biological and behavioral mechanisms may further explain why sleep quality and daytime sleepiness relate differently to injury risk in males and females. Physiologically, females generally exhibit greater sleep need, higher slow-wave activity, and greater sensitivity to sleep fragmentation, which may amplify the functional consequences of poor sleep quality on cognitive control, proprioception, and emotional regulation. Hormonal factors, including fluctuations in estrogen and progesterone across the menstrual cycle, also influence sleep architecture, pain sensitivity, and neuromuscular function, potentially increasing vulnerability to musculoskeletal strain when sleep is disrupted [49].

Males, in contrast, tend to accumulate more sleep debt, maintain more irregular sleep schedules, and display greater variability in circadian timing, all of which may compound the effects of daytime sleepiness on risk-taking behaviors and attentional lapses. Behavioral factors such as higher engagement in intense physical activity, greater training load variability, and increased propensity for impulsive or high-risk movement strategies may also magnify the impact of sleep-related impairments in males. Additionally, sex differences in stress reactivity and coping strategies may influence how sleep disturbances translate into neuromuscular fatigue or biomechanical compensation patterns [50].

Together, these physiological, hormonal, and behavioral mechanisms provide a plausible explanation for why poor sleep quality had a stronger and more independent association with injury in females, whereas males exhibited a descriptive tendency toward interaction between sleep quality and daytime sleepiness.

The additive interaction (synergy) analysis provided further insight into how SQ and EDS combine to influence injury risk. In males, RERI, AP, and S did not support supra-additivity, and the RR structure indicated near-multiplicative rather than additive interaction. In females, the qualitative supra-additive structure—although weak—suggested that the combined effect of poor sleep quality and daytime sleepiness may be more harmful than either factor alone. Despite the small effect sizes, this pattern is noteworthy because additive interactions have direct implications for public health interventions: they identify subgroups for whom dual exposures confer greater absolute risk meaningfully [51,52]. However, wide confidence intervals emphasize the need for larger samples to achieve more precise estimates.

Beyond sleep-related determinants, musculoskeletal injury in physically active young adults is widely recognized as a multifactorial phenomenon shaped by an interplay of intrinsic risk factors, including anthropometric characteristics, body composition, postural alignment, and the quality of fundamental movement patterns. In the present study, sleep-related variables explained only around 3–4% of the variance in injury occurrence (Nagelkerke R^2^ ≤ 0.037 in females; ≤0.012 in males), indicating a modest contribution compared with other well-established intrinsic determinants. This is consistent with the broader literature on sports injuries, which emphasizes the multifactorial nature of injury susceptibility, including anthropometric and body-composition indices (e.g., BMI, FMI), musculoskeletal alignment, and neuromuscular control as key intrinsic risk factors.

Anthropometric and body composition indices typically demonstrate considerably stronger relationships with injury risk. Previous work in physically active populations has shown that indices such as BMI, SMI, FMI and MFR achieve AUC values of 0.60–0.63, corresponding to approximately 10–20% explanatory contribution to injury risk—substantially exceeding the predictive capacity of sleep measures observed here [34,53,54]. Generally, evidence from those works indicates that higher BMI and weight are risk factors for musculoskeletal injuries, particularly ankle sprains. However, it remains unclear whether BMI or FMI is more important, although recent results confirm the greater importance of FMI [34]. In comparison to the very low predictive contribution of sleep-related factors—where PSQI and ESS explained at most 3–4% of the discrimination beyond chance (AUC ≈ 0.51–0.56)—anthropometric indices demonstrated substantially greater predictive capacity, with AUC values ranging from 0.57 to 0.63, corresponding to 14–28% predictive signal above chance [34,55]. This indicates that body mass– and fat-related indices contribute 5–7 times more to injury discrimination than sleep variables, underscoring the dominant role of somatic intrinsic risk factors relative to sleep-related determinants in physically active young adults.

Further intrinsic determinants include postural alignment and spinal morphology, which influence load distribution and joint mechanics during physical activity. Misalignment of the vertebral column—such as excessive thoracic kyphosis or altered lumbar curvature—can modify movement efficiency and predispose individuals to overload syndromes. Prior work has shown sport-specific differences in sagittal-plane spinal curvature, including crossfit-related modifications of spinal posture that may influence loading patterns and musculoskeletal vulnerability [56]. These biomechanical factors likely account for a substantially larger share of injury variance than sleep alone. Previous work has shown that abnormal body-mass distribution and altered postural alignment can increase mechanical loading and tissue stress during physical activity [56,57]. The interaction between frontal plane alignment and body mass significantly affects dynamic knee joint loading in knee osteoarthritis patients, with alignment having a greater impact on knee load in those with higher body mass [58]. Translating these mechanical effects into estimated injury susceptibility, such values imply that lower-limb alignment and posture may explain approximately 30–50% of the intrinsic biomechanical contribution to injury risk, a magnitude far exceeding the 3–4% predictive influence attributable to sleep-related factors in the present study.

Similarly, movement pattern quality—typically assessed with the Functional Movement Screen (FMS)—is one of the strongest intrinsic predictors of injury risk. Across diverse athletic cohorts, FMS demonstrates AUC values ranging from 0.56 to 0.74, corresponding to 10–25% of explained variance in injury susceptibility. High-quality studies report that FMS scores below threshold (<14) substantially increase injury risk [59,60], while meta-analyses confirm consistent though moderate predictive power [61,62]. The mechanism can be explained, among other things, by movement quality deficits—including asymmetries, poor neuromuscular coordination and limited mobility, which have been linked to reduced agility and suboptimal motor performance in sport populations [63] and their functional relevance.

Taken together, these data demonstrate that while sleep quality has a measurable—but modest—association with injuries, its contribution is far smaller than that of somatic, postural and neuromuscular intrinsic factors. Therefore, sleep should be understood as an auxiliary, not primary, determinant of injury susceptibility in physically active young adults.

Hypothesis evaluation. In relation to the hypotheses stated in the Introduction, the results provide partial and differential support. H1 was partially confirmed: poor sleep quality was independently associated with higher injury occurrence—particularly among females—whereas excessive daytime sleepiness did not show an independent effect. H2 was not confirmed, as neither multiplicative nor additive interaction yielded statistically robust evidence of synergy, although males displayed a descriptive tendency toward interaction. H3 was confirmed because the dependence structure linking sleep quality, daytime sleepiness, and injury occurrence differed clearly between males and females, with males showing a descriptive interaction pattern and females demonstrating a strictly additive structure. H4 was also confirmed, as sleep-related factors explained only 1–4% of the variance in injury risk, indicating a modest contribution relative to well-established intrinsic determinants such as body composition, postural alignment, and movement quality. Taken together, the findings suggest that sleep quality plays a small but measurable role in injury occurrence, whereas daytime sleepiness contributes minimally beyond its correlation with sleep quality.

Strengths. This study’s strengths include its multi-method analytical framework, which integrated chi-square tests, hierarchical log-linear modeling, and additive interaction metrics (RERI, AP, S). This approach allowed us to separate classical multiplicative interaction from risk-based additive synergy and to identify subtle sex-specific patterns that would likely remain undetected using a single analytic method. The large and relatively homogeneous group of physically active university students (*n* = 418) further strengthened the internal validity of subgroup analyses.

Limitations. However, several limitations should be considered. The cross-sectional design prevents causal inference, and therefore, the observed associations cannot establish temporal ordering or causality. Another important limitation is that all measures—both sleep characteristics (PSQI, ESS) and injury occurrence—were based on self-report, which may introduce recall bias or misclassification and affect the precision of the findings. Although self-reported sleep instruments are widely used, future work should incorporate objective measures such as actigraphy.

The analyses of multiplicative and additive interaction are exploratory, as the sample size—particularly in sex-stratified subgroups—limited the precision of interaction estimates, resulting in wide confidence intervals. In addition, the study did not include key potential confounders such as mental health indicators (e.g., depression or stress), chronic injury history, or training load, all of which may influence both sleep characteristics and injury risk. The absence of these variables restricts the ability to fully account for alternative explanatory pathways.

Finally, the findings apply specifically to physically active students from a single university. As such, they cannot be generalized to students with lower activity levels, individuals from other academic settings, or athletic populations with different training characteristics. This limits the external validity of the results.

Practical implications. These findings should be interpreted with caution, as sleep-related factors explained only 1–4% of the variance in injury occurrence. Therefore, the practical impact of sleep characteristics on injury prevention is modest. Sleep screening may still be useful as an auxiliary component of broader injury-prevention strategies, particularly in identifying individuals with markedly poor sleep quality; however, it should not be considered a primary predictive tool. For females, improving sleep quality may offer small incremental benefits, whereas for males, the descriptive interaction between SQ and EDS suggests that concurrent disturbances might warrant attention. Overall, practical applications should focus primarily on more influential intrinsic risk factors, with sleep functioning as a supplementary consideration rather than a central determinant.

Future directions. Future research should employ longitudinal designs to clarify the temporal relationship between sleep disturbances and injury occurrence. A longitudinal approach is also essential to confirm the present findings and to evaluate both multiplicative and additive interaction effects with greater statistical precision, overcoming the limitations inherent to cross-sectional data. Objective sleep measures are needed to validate the sex-specific interaction patterns found here. Further work should also examine moderators such as chronotype, stress, academic load, and training intensity. Larger samples will improve the precision of interaction metrics, particularly in females, where supra-additive tendencies warrant further investigation. Advanced modeling approaches (e.g., machine learning, non-linear risk functions) may uncover thresholds or nonlinearities in sleep–injury relationships not detectable with conventional models.

## 5. Conclusions

This study demonstrates clear sex-specific patterns in how sleep quality and daytime sleepiness relate to injury occurrence in physically active university students. Poor sleep quality was the primary sleep-related predictor of injuries—particularly among females—while daytime sleepiness showed only weak and inconsistent effects. Interaction patterns diverged across sexes: males exhibited a descriptive tendency toward classical interaction between SQ and EDS, whereas females showed a strictly additive structure. Despite these differences, the overall explanatory power of sleep-related factors was low, indicating that sleep contributes only modestly to injury risk. Given the cross-sectional design, these findings represent associations only and cannot establish causal relationships between sleep characteristics and injury. These findings highlight the need to consider sleep characteristics alongside a broader set of intrinsic risk factors—such as body composition, postural alignment, and movement quality—when assessing injury vulnerability in young adults.

## Figures and Tables

**Table 1:** Baseline characteristics of the study participants (*n* = 418).

Variable			Males			Females			
	Mean	95% CI Lower	95% CI Upper	SD	Mean	95% CI Lower	95% CI Upper	SD	*p*
Age [y]	20.73	20.61	20.85	0.85	20.56	20.46	20.65	0.74	**0.025**
Body height [cm]	182.19	181.20	183.18	7.10	168.17	167.37	168.97	6.01	**<0.001**
Body weight [kg]	79.63	78.25	81.01	9.87	60.86	59.65	62.06	9.05	**<0.001**
BMI [kg/m^2^]	23.97	23.62	24.32	2.48	21.49	21.12	21.85	2.71	**<0.001**
IPAQ [MET-min/week]	3608.2	3418.5	3797.9	1356.9	3019.8	2886.5	3153.1	1000.9	**<0.001**
PSQI [pts]	4.42	4.13	4.72	2.10	4.26	4.00	4.53	1.98	0.431
ESS [pts]	12.15	11.50	12.79	4.60	11.82	11.18	12.45	4.78	0.476

BMI—body mass index; IPAQ—International Physical Activity Questionnaire, tool used to measure and compare physical activity levels in populations; PSQI—Pittsburgh Sleep Quality Index, indicates the sleeping quality (SQ); ESS—Epworth Sleepiness Scale, indicates the daytime sleepiness revealing the exceeded daytime sleepiness (EDS); SD—standard deviations; CI—confidence interval; statistical significance is marked in bold font.

**Table 2:** Frequency distribution of sex, sleep quality, and daytime sleepiness categories, with corresponding proportions of participants reporting at least one injury (Injury = 1). Values are presented as counts (*n*) and percentages (%).

Factor	Category	*n*	%	Injury (%)
Sex	Males	199	47.6	56.8
	Females	219	52.4	46.6
Sleep quality (SQ)	Good	280	67.0	47.5
	Poor	138	33.0	59.4
Excessive Daytime Sleepiness (EDS)	Normal	138	33.0	47.1
	Sleepiness	280	67.0	53.6

**Table 3:** Associations between sex, sleep variables, and injury occurrence (2 × 2 contingency analyses).

Comparison	χ^2^	Fisher’s *p*	φ	OR (95% CI)
Sex × INJ	0.57	0.453	0.04	1.16 (0.79–1.69)
Sex × SQ	0.35	0.556	0.03	1.12 (0.78–1.61)
Sex × EDS	0.10	0.816	0.02	1.08 (0.76–1.53)
SQ × INJ (all)	**4.76**	**0.029**	0.11	1.60 (1.05–2.45)
EDS × INJ (all)	1.24	0.265	0.06	1.27 (0.83–1.95)
SQ × INJ (males)	0.64	0.424	0.06	1.30 (0.68–2.48)
SQ × INJ (females)	**5.39**	**0.020**	0.16	1.98 (1.11–3.56)
EDS × INJ (males)	0.09	1.000	0.03	1.11 (0.58–2.15)
EDS × INJ (females)	2.09	0.148	0.10	1.47 (0.86–2.51)

INJ = injury occurrence (0 = no injury, 1 = injury); SQ = sleep quality (0 = good, 1 = poor); EDS = excessive daytime sleepiness (0 = normal, 1 = excessive). *p*-values from Fisher’s exact tests are reported. Odds ratios express the relative likelihood of injury (INJ = 1) between respective categories. Statistically significant comparisons are marked in bold. Underlined values are marked as trends.

**Table 4:** Log-linear model comparison for the association between sleep quality, daytime sleepiness, and injury occurrence (SQ × EDS × INJ; *n* = 418) in the male group.

Model	Deviance (G^2^)	df	ΔG^2^	Δdf	*p* (GOF)
Main effects	20.79	4	-	-	<0.001
Two-way interactions (SQ × INJ, EDS × INJ, SQ × EDS)	2.42	1	18.37	3	0.119
Three-way interaction (SQ × EDS × INJ)	0	0	2.42	1	0.119

INJ = injury occurrence (0 = no injury; 1 = injury); SQ = sleep quality (0 = good; 1 = poor); EDS = excessive daytime sleepiness (0 = normal; 1 = excessive). GOF = goodness-of-fit test relative to the saturated model.

**Table 5:** Log-linear model comparison for the association between sleep quality, daytime sleepiness, and injury occurrence (SQ × EDS × INJ; *n* = 418) in the female group.

Model	Deviance (G^2^)	df	ΔG^2^	Δdf	*p* (GOF)
Main effects	27.08	4	-	-	<0.001
Two-way interactions (SQ × INJ, EDS × INJ, SQ × EDS)	0.011	1	27.07	3	0.917
Three-way interaction (SQ × EDS × INJ)	0	0	0.011	1	0.917

INJ = injury occurrence (0 = no injury; 1 = injury); SQ = sleep quality (0 = good; 1 = poor); EDS = excessive daytime sleepiness (0 = normal; 1 = excessive). GOF = goodness-of-fit test relative to the saturated model.

**Table 6:** Additive interaction (Poisson w/robust SE; adjusted for sex, age, and BMI).

Sex	RR_10_	RR_01_	RR_11_	RERI	AP	S
Males	0.64	0.90	1.09	0.55	0.50	−0.20
Females	1.32	1.22	1.61	0.07	0.04	1.12

RR_10_ (Poor SQ only), RR_01_ (Excessive EDS only), and RR_11_ (Both factors); RERI—Relative Excess Risk due to Interaction; AP—Attributable Proportion due to Interaction; S—Synergy Index.

**Table 7:** Logistic regression models for injury occurrence with sleep variables in males and females.

Sex	Model	Nagelkerke R^2^	LR χ^2^ (df) vs. Null	*p* (LR)	AIC	BIC
Males	SQ	0.006	0.94 (1)	0.33	275.26	281.85
	EDS	~0.000	0.01 (1)	0.93	276.19	282.78
	SQ + EDS	0.007	0.98 (2)	0.61	277.22	287.10
	SQ × EDS	0.012	1.79 (3)	0.62	278.40	291.58
Females	SQ	0.037	6.15 (1)	**0.013**	300.42	307.20
	EDS	0.016	2.69 (1)	0.10	303.88	310.66
	SQ + EDS	0.043	7.11 (2)	**0.029**	301.46	311.62
	SQ × EDS	0.043	7.20 (3)	0.066	303.37	316.93

Significant results are in bold, and trend is underlined (*p*~0.1). SQ—sleep quality; EDS—Excessive daytime sleepiness; SQ × EDS—interaction term.

## Data Availability

The data presented in this study are available on request from the author.
